# Molecular Genetic Approach and Evaluation of Cardiovascular Events in Patients with Clinical Familial Hypercholesterolemia Phenotype from Romania

**DOI:** 10.3390/jcm10071399

**Published:** 2021-03-31

**Authors:** Cristiana-Elena Vlad, Liliana Georgeta Foia, Roxana Popescu, Ioana Popa, Ruxandra Aanicai, Delia Reurean-Pintilei, Vasilica Toma, Laura Florea, Mehmet Kanbay, Adrian Covic

**Affiliations:** 1 Faculty of Medicine, “Grigore T Popa” University of Medicine and Pharmacy, University Street, No 16, 700115 Iasi, Romania; vladcristiana@gmail.com (C.-E.V.); roxana.popescu2014@gmail.com (R.P.); ioanap93@yahoo.com (I.P.); ruxi03@yahoo.co.uk (R.A.); drdeliapintilei@gmail.com (D.R.-P.); vasilicatoma40@yahoo.com (V.T.); lflorea68@yahoo.com (L.F.); accovic@gmail.com (A.C.); 2 Department of Nephrology-Internal Medicine, “Dr. C.I. Parhon” Clinical Hospital, Carol I Street, No 50, 700503 Iasi, Romania; 3 Department of Biochemistry, “Sf. Spiridon” Clinical County Hospital, Independentei Street, 700111 Iasi, Romania; 4 Department of Medicine, Division of Nephrology, Koc University School of Medicine, 34450 Istanbul, Turkey; mkanbay@ku.edu.tr

**Keywords:** familial hypercholesterolemia, cardiovascular events, pathogenic mutations, molecular genetic

## Abstract

This study identifies the genetic background of familial hypercholesterolemia (FH) patients in Romania and evaluates the association between mutations and cardiovascular events. We performed a prospective observational study of 61 patients with a clinical diagnosis of FH selected based on Dutch Lipid Clinic Network (DLCN) and Simon Broome score between 2017 and 2020. Two techniques were used to identify mutations: multiplex ligation-dependent probe amplification (MLPA) and Sanger sequencing. The mutation rate was 37.7%, i.e., 23 patients with mutations were identified, of which 7 subjects had pathogenic mutations and 16 had polymorphisms. Moreover, 10 variants of the low-density lipoprotein receptor (*LDLR*) gene were identified in 22 patients, i.e., one variant of the proprotein convertase subtilisin/kexin type 9 (*PCSK9*) gene in six patients, and one variant of the apolipoprotein B (*APOB*) gene in three patients. Of the *LDLR* gene variants, four were *LDLR* pathogenic mutations (c.81C > G, c.502G > A, c.1618G > A mutations in exon 2, exon 4, exon 11, and exon 13–15 duplication). The *PCSK9* and *APOB* gene variants were benign mutations. The pathogenic *LDLR* mutations were significant predictors of the new cardiovascular events, and the time interval for new cardiovascular events occurrence was significantly decreased, compared to FH patients without mutations. In total, 12 variants were identified, with four pathogenic variants identified in the *LDLR* gene, whereas 62.3% of the study population displayed no pathological mutations.

## 1. Introduction

Familial hypercholesterolemia (FH) (Online Mendelian Inheritance in Man-OMIM 143890) is an autosomal dominant genetic pathology, frequently caused by pathogenic variants of the low-density lipoprotein receptor (LDLR), apolipoprotein B (APOB), and the proprotein convertase subtilisin/kexin type 9 (PCSK9) [1,2,3,4,5,6,7,8,9]. In Europe, the FH frequency with the heterozygous form varies from 1:200 to 1:500 individuals, being extremely rare in the homozygous form, which ranges from 1:300,000 to 1:1.000,000 in the general population [4,5,7,10]. These pathological variants trigger elevated low-density lipoprotein cholesterol (LDL–C) levels, leading to accelerated atherosclerosis and atherosclerotic cardiovascular disease (ASCVD) [1,5,6,7,8,11]. Atherosclerosis is a complex multifactorial disorder consisting of the chronic inflammatory response, which then causes plaque formation in the intima and media of medium and large arteries [12]. Hypercholesterolemia causes the proliferation of hematopoietic and progenitor cells, leading to leukocytosis and increased atherosclerosis [13]. Thereby, patients with high LDL–C values and low high-sensitivity C-reactive protein (hsCRP) levels have a lower risk of stroke, coronary heart disease (CHD), and death from CHD, compared to those with high levels of LDL–C and hsCRP [13]. The clinical and biological diagnosis of FH is based on the Dutch Lipid Clinic Network (DLCN) score, Simon Broome, and US MedPed [6].

The low-density lipoprotein receptor (LDLR) is a cell surface glycoprotein that mediates the specific binding and uptake of apoB-100 lipoproteins by receptor-mediated endocytosis [14]. The *LDLR* gene locus is located on chromosome 19p13.1-13.3, with 18 exons and 17 introns, encoding a mature protein of 839 amino acids and including a signal sequence of 21 amino acids with a series of five discrete major structural domains [6,7,8,10,14]. *LDLR* gene mutations are the most common FH cause (90–95%), whereas *APOB* mutations account for only 3–6%, and mutations in *PCSK9* are found in less than 1–3% of patients [2,15]. The diversity of the underlying variants is wide; over 2000 variants have been documented for *LDLR*, with monogenic and polygenic forms and increased FH penetrance, yet the expressivity varies depending on the nature of the mutation [3,6,7,15]. Each country has its specific spectrum of *LDLR* mutations [3]. In addition, the mutations located in the *APOB* gene alter the functional activity of the apolipoprotein B, decreasing its binding to LDLR and reducing the clearance of LDL particles and the accumulation of LDL–C [16]. PCSK9 is a serine protease of the subtilase family, secreted primarily by the liver and kidney via sterol regulatory element-binding protein 2 (SREBP-2) modulation and, by a second transcription factor, the hepatocyte nuclear factor 1 (HNF1) [17,18,19]. The PCSK9 plasma levels are closely related to nutritional and hormonal status (e.g., hepatic glucagon receptor signaling) and diurnal rhythm [19]. The azacytidine activation associated with deacetylase sirtuin 1 contributes to reduced PCSK9 secretion, confirming the important role of epigenetic regulation by DNA methylation and histone acetylation [19]. PCSK9 is a novel therapeutic target for familial hypercholesterolemia designed for lowering cardiovascular risk (PCSK9 is observed in carotid atherosclerotic lesions) through monoclonal antibodies and small interfering RNA [19,20]. PCSK9 determines the degradation of LDLR and inhibits receptor recycling in the hepatocyte membrane [19,20,21].

The early diagnosis and initiation of adequate lipid-lowering drugs are based on a proper understanding of the molecular mechanisms of this disease and contribute to a significant reduction in cardiovascular morbidity and mortality [4,8]. The cascade screening system has been used in many European countries (e.g., the Netherlands, Norway, Iceland, Switzerland, the United Kingdom, and Spain) as an effective way to identify FH patients [4,6]. However, in some European countries (e.g., Romania), FH is still underdiagnosed and mistreated [4,6]. In Romania, the genetic characterization of FH has not been explored adequately because there are no studies on the molecular aspects of this pathology. Our prospective observational study included patients with FH, with the following objectives: (a) to identify the mutation in order to establish the genetic background of FH patients in Romania and (b) to evaluate the association between the identified mutations and the cardiovascular events.

## 2. Materials and Methods

### 2.1. Patient Recruitment

The study was designed as an observational, prospective, three-year study (October 2017 to October 2020) in three referral centers from the northeastern region of Romania, which includes eight counties and a population of over 3,980,000 inhabitants.

The study population included 980 patients with dyslipidemia who were identified between September 2016 and October 2017, and 61 patients meeting the following inclusion criteria: (a) subjects with full mental capacity who signed the informed consent form and (b) men and women aged over 18 years. The DLCN score above 3 and the Simon Broome criteria (probable or possible FH) represented two important selection tools for the patients with a clinical diagnosis of FH. These criteria included the following elements: identification of a family history of hypercholesterolemia or cholesterol deposits in vascular and extravascular tissues; setup of a personal history of early onset of coronary, cerebrovascular, and peripheral vascular diseases; clinical observations regarding the presence of either xanthomas, xanthelasma and/or arcus cornealis; biological identification of total cholesterol (TC) > 300 mg/dL, LDL–C > 190 mg/dL without treatment, or >100 mg/dL following treatment with maximum doses of statins (40 mg rosuvastatin, 80 mg atorvastatin), in combination with ezetimibe.

Exclusion criteria included the following: subjects lacking discernment or those who refused to sign the informed consent; patients under the age of 18; pregnant and breastfeeding women; subjects with severe physical disabilities, dementia, neoplasms, and other causes of secondary hypercholesterolemia (uncontrolled diabetes, nephrotic syndrome, hypothyroidism, drug-induced dyslipidemia) [22].

### 2.2. Clinical and Biological Evaluation of FH Patients

The patients included in the study were coded with the letter H and the corresponding ID number. The medical history revealed that certain patients had received antihypertensive medication (those with BP >140/90 mmHg) or oral antidiabetic medication (those diagnosed with type 2 diabetes), which was allowed throughout the study according to the specialist doctors’ prescription.

The study included patients with a DLCN score >3 for the FH population [23,24]. The reference values of the DLCN score were as follows: 3–5 points highlighted possible FH, 6–7 points indicated probable FH, while over 8 points indicated definite FH [23,24]. The other score, namely, the Simon Broome score, flagged the presence of possible, probable, or definitive FH [23,24].

Laboratory tests included values at baseline, and at 12, 24, and 36 months; total cholesterol mg/dL, LDL–C mg/dL, high density cholesterol lipoprotein (HDL–C) mg/dL, triglycerides (TG) mg/dL, blood glucose (mg/dL), and high-sensitivity C-reactive protein (hsCRP) mg/dL were measured by spectrophotometric assay (Architect c8000—Abbott Laboratory, Chicago, IL, USA).

### 2.3. Evaluation of the New Cardiovascular Events

Atherosclerotic cardiovascular disease (ASCVD) was defined as a history of one of the following diseases, as identified in the medical records: coronary heart disease (CHD) (with particularities such as acute coronary syndrome, myocardial infarction (MI), stable angina, coronary revascularization, ischemic stroke, or transient ischemic attack) and peripheral artery disease (PAD) [25].

Further explorations for cardiovascular evaluation included the following:

- electrocardiogram (ECG) for ischemic changes assessment;
- ankle–brachial index (ABI) measurement with a sphygmomanometer and a portable ultrasonography device for determining sounds that detect systolic blood pressure in the lower limbs; the reference ABI values were between 0.9 and 1.3;
- echocardiography (Siemens Acuson CV70 Cardiac Vascular Ultrasound Machine), highlighting left ventricular (LV) wall motion abnormalities and ejection fraction values, important predictors of left ventricular systolic dysfunction;
- measurement of carotid intima–media thickness (cIMT) (at the levels of the carotid bifurcation, internal, external, right and left carotid arteries) by using Siemens Acuson CV70 Cardiac Vascular Ultrasound Machine, B-mode and color Doppler ultrasound (5–10 MHz). The average of the cIMT (the average of the six quantified segments) was also recorded. The reference cIMT values were under 0.9 mm [26,27].

### 2.4. Evaluation of the Mutations in the LDLR, APOB, and PCSK9 Genes

#### 2.4.1. DNA Genomic Extraction

DNA was extracted from 3 mL of peripheral blood samples stored with EDTA agent, using Wizard Genomic DNA Purification Kit (Promega Corp., Madison, WI, USA).

#### 2.4.2. MLPA (P062, LDLR MLPA Kit, MRC Holland, Amsterdam, Netherlands)

The probe mix P062 LDLR was used for the deletion of or duplication in the LDLR genes. The multiplex ligation-dependent probe amplification (MLPA) analysis was performed according to the manufacturer’s protocol. The genomic DNA was denatured and hybridized with P062 probes at 60 °C for approximately 17 h. The PCR amplification was performed after 15 min ligation at 54 °C, using Cy5 labeled primers. Fluorescent amplification products were separated based on their length by capillary electrophoresis in a CEQ 8000 GeXP Genetic Analysis System (Beckman Coulter, Brea, CA, USA), and the results were analyzed using the Coffalyser.NET program (MRC-Holland, Amsterdam, The Netherlands). The probe ratios of deletion and duplication were fixed at 0.7 and 1.3, respectively [28].

#### 2.4.3. Sanger Sequencing

The LDLR gene coding region and intron-exon boundaries were sequenced bi-directionally for all the patients. From the coding region were evaluated the exon 7 for PCSK9 and exon 26 for APOB.

Approximately 125 ng genomic DNA was amplified in a 25 µL reaction volume, on Sensoquest Thermocycler (Sensoquest, Göttingen, Germany), using GoTaq^®^ G2 Hot Start Master Mix (Promega, Madison, WI, USA). The PCR conditions were initial denaturation (10 min at 95 °C), followed by 35 cycles of denaturation (30 s at 94 °C), annealing varying between 55 and 66 degrees depending on the amplified fragments and elongation (60 s at 72 °C), with a final elongation at 72 °C for 5 min. The sequencing was performed using *GenomeLab DTCS*-*Quick Start Kit* (Beckman Coulter, Brea, CA, USA) in a 10 µL reaction volume. The PCR and sequencing products were purified with the Agencourt system (Beckman-Coulter, USA), Agencourt AMPure XP, and Agencourt Cleanseq^®^ system, respectively. The final products were subsequently separated by capillary electrophoresis on CEQ 8000 GeXP Genetic Analysis System (Beckman-Coulter) [28]. Sequences were analyzed using MegaX software and were compared with the corresponding reference sequences, namely, NM_000527.5 for *LDLR*, NM_000384.3 for *APOB*, and NM_174936.3 for *PCSK9*. The variants were verified in Mutation taster, ClinVar, and PolyPhen for predicting the functional effect of DNA sequence alterations [28,29].

### 2.5. Statistical Analysis

The data of the FH patients were introduced into a database and processed by means of the statistical functions of the SPSS version 20.0 system. One-sample Kolmogorov–Smirnov for normal distribution tests were performed, with the data being calculated as mean and standard deviation (SD) for normal distribution variables, percent for categorical variables by using a frequency test, and median and interquartile range (IQR) for continuous variables with asymmetrical distribution. Bivariate correlation analysis was performed between the scale variables, using the Spearman correlation coefficient. To evaluate the associations between nominal variables, specific association coefficients were used (Cramer’s, Phi, contingency coefficient, chi square (χ^2^)). Comparative analyses between the pathological history, clinical, and paraclinical history according to mutations were achieved for the values that did not meet the criteria of normal homogeneity. The normal distribution was performed for nonparametric tests, i.e., Mann–Withney U sample, Wilcoxon signed rank, Kruskall–Wallis H test, and Friedman test. Survival free of ASCVD, during follow up and according to mutations, was estimated using the Kaplan–Meier method. The duration of the follow up was calculated from the date of inclusion in the study to the date of the occurrence of cardiovascular events. Multiple logistic regression analysis was applied to detect the independent factors for cardiovascular events. The *p*-value < 0.05 was considered statistically significant.

## 3. Results

### 3.1. The Genetic Spectrum of FH in Romania

The study group included 61 patients (6.2% of all patients examined), with a mean age of 48.5 ±12.5 years old, all subjects being Caucasian, with a higher proportion of women compared to men (63.9% versus 36%). Moreover, 36.1% of the patients had ASCVD history (Table 1). The laboratory results recorded TC 315 ± 56 mg/dL; LDL–C 254.2 ± 53 mg/dL; HDL–C 45.8 ± 18 mg/dL; and TG 174.4 ± 92 mg/dL (for all patients), whereas the lipid profile (LDL–C, HDL–C, TG) did not differ according to mutations (Table 1). Furthermore, the FH patients who had pathogen/likely pathogen mutations had significantly increased TC levels and high DLCN score values, compared to mutation-free patients with a clinical diagnosis of FH and FH patients with benign/likely benign mutations (Table 1). Moreover, FH patients with pathogenic mutations had significantly increased TC levels and high values of DLCN score, compared to patients without mutations. The number of patients with pathogenic mutations, male, active smokers, with high blood pressure, cardiovascular history, obesity, and diabetes was reduced, compared to the number of subjects without mutations (Table 1). The same results were observed in patients with benign variants, compared to subjects without mutations. At baseline, all the patients had lipid-lowering therapies (about one year of treatment prior to inclusion in the study), the most frequent being the statin monotherapy (36.1%), followed by associations between statin and ezetimibe, statin and fenofibrate, and their triple combination (Table 1). There were no significantly different percentages between patients with benign/pathogenic mutations versus patients without mutations who received lipid-lowering treatments.

**Figure 1:**
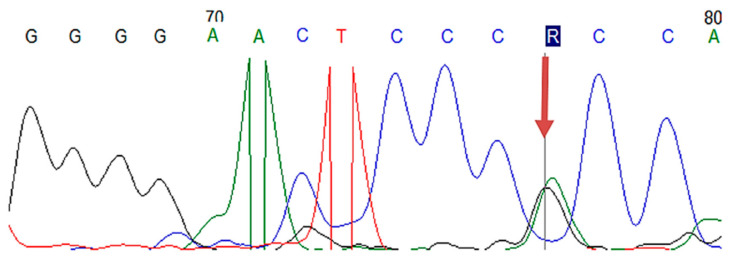
The Sanger sequencing electropherogram in patients H19, H46, and H53: pathogen mutation of *LDLR* in exon 11 (c.1618G > A).

**Figure 2:**
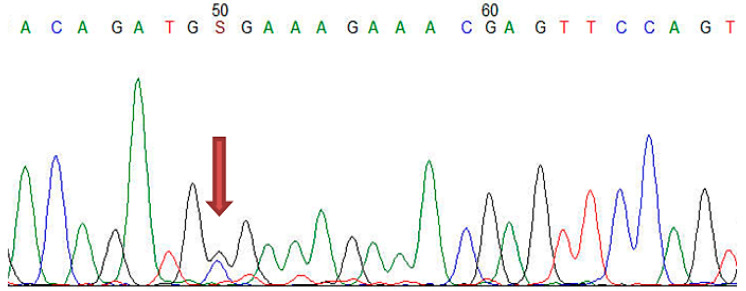
The Sanger sequencing electropherogram in patients H18 and H42: pathogen mutation of *LDLR* in exon 2 (c.81C > G).

**Figure 3:**
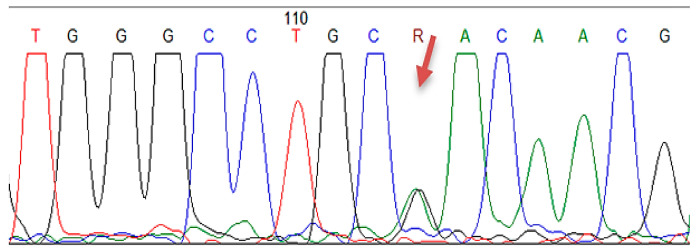
The Sanger sequencing electropherogram in patient H54: pathogen mutation of *LDLR* in exon 4 (c.502G > A).

**Figure 4:**
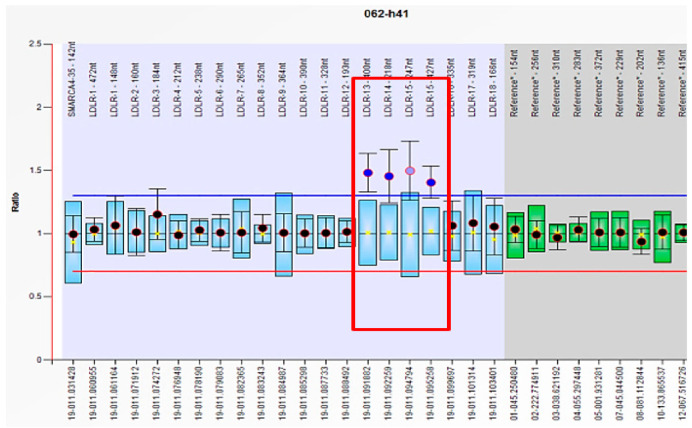
Multiplex ligation-dependent probe amplification (MLPA) method shows the duplication (ratio~1.5) in *LDLR* exons 13–15 in patient H41-exon numbers were shown above as “LDLR-number of exons” (the LDLR exon numbering uses the RefSeq transcript NM_000527.4). The reference probes were included for normalized probe-signal ratio. The arbitrary border (upper and lower) was placed ±0.3 from the reference sample median of probes.

In the study group with 61 FH patients, mutations in *LDLR, APOB, PCSK9* genes were analyzed by MLPA and Sanger sequencing, revealing pathological results in 23 cases, with a mutation detection rate of 37.7%. Among these subjects, 16 patients had benign/pathogenic variants in *LDLR* gene, 4 patients in *LDLR* and *PCSK9* genes, 1 subject in *LDLR* and *APOB* genes, 1 subject in *APOB* and *PCSK9* genes, and 1 patient in all candidate genes (*LDLR*, *APOB, PCSK9*) (Table 2 and Table S1). Regarding the clinical significance of FH-associated mutations in ClinVar, respectively, Leiden Open Source Variation Database (LVOD), out of the 23 patients identified with mutations, seven subjects had pathogenic/likely pathogenic mutations and the others had either benign/likely benign mutations or conflicting interpretations (Table 2 and Table S1). The pathogenic/likely pathogenic *LDLR* mutations were detected in three patients having the same mutation in *LDLR* exon 11 (c.1618G > A homozygous) (Figure 1, Table 2 and Table S1), in two patients bearing the same mutation in *LDLR* exon 2 (c.81C > G heterozygous) (Figure 2, Table 2 and Table S1), one patient displaying mutation in *LDLR* exon 4 (c.502G > A heterozygous) (Figure 3, Table 2 and Table S1), and one patient showing duplication in exon 13–15 (c.(1845 + 1_1846-1)_(2311+1_2312-1)dup)) (Figure 4; Table 2 and Table S1). In this study, the c.1026A > G in the *PCSK9* gene and the c.10740C > T in the *APOB* gene were benign/likely benign variants (Table 2 and Table S1).

**Figure 5:**
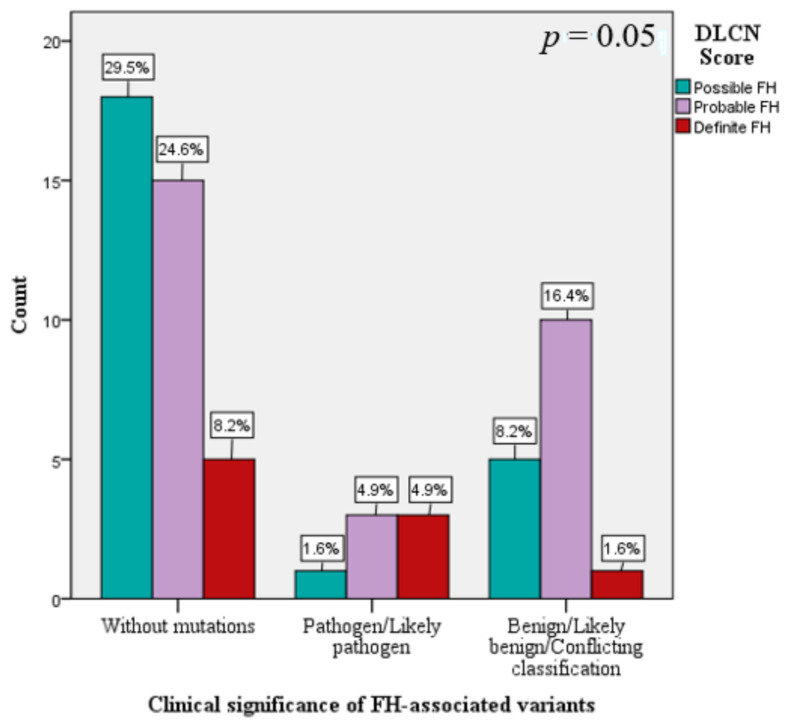
The frequency of mutations and DLCN score.

According to the DLCN score, the FH patients without mutation had increased frequencies of probable FH and of defined FH, compared to FH patients with benign/likely benign/conflicting classification or pathogen/likely pathogen mutations (χ^2^(2) = 5.7, *p* = 0.05) (Figure 5). Depending on the DLCN score, the subjects with probable FH had 4.9% pathogenic/likely pathogenic mutations and 16.4% benign/ likely benign mutations, while the individuals with definite FH had 4.9% pathogenic/likely pathogenic mutations and 1.6% benign/likely benign mutations (Figure 5).

### 3.2. The New ASCVD in Patients with FH Based on LDLR, APOB, and PCSK9 Mutations

The seven patients with pathogenic mutations advertised new cardiovascular events as follows: three patients with c.1618G > A in homozygous state had the acute coronary syndrome, two with c.81C > G in the heterozygous state had a stroke and peripheral artery disease (PAD) and one with c.502G > A and dup ex13–15 had a stroke (Table S1). Additionally, among the 16 patients with benign/likely benign mutations, only eight had new ASCVD: three patients experienced a stroke, three had PAD, and two patients had acute coronary syndrome (Table S1).

Patients with pathogenic/likely pathogenic mutations and with ASCVD had elevated TC, LDL–C levels throughout the follow up, with cIMT values increased at 12 and 36 months, whereas the ejection fraction (EF) and HDL–C values were significantly declined at 36 months, compared to patients with benign mutations and ASCVD or mutation-free subjects with ASCVD (Table 3). The FH patients with ASCVD but no mutations recorded elevated hsCRP levels throughout the follow-up period, compared to FH patients with ASCVD and mutations. Moreover, ankle–brachial index (ABI) values were decreased in FH patients with ASCVD and benign mutations, compared to patients with ASCVD without mutations (Table 3).

Furthermore, in FH patients, following the multiple regression, pathogenic/likely pathogenic mutations were significant predictors of the new cardiovascular events (OR = 4.81, 95% CI: 1.26–18.32, *p* = 0.02) (Table 4). Interestingly, lipid-lowering drugs did not act as a protective factor for the new cardiovascular disease in the case of these patients (*p* = 0.72) (Table 4).

**Figure 6:**
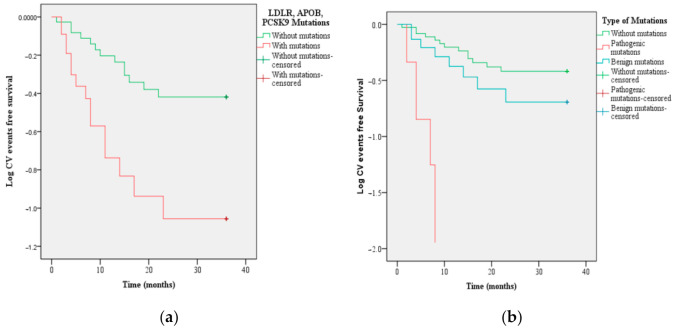
Kaplan-Meier for ASCVD depending on (**a**) *LDLR*, APOB, and PCSK9 mutations and (**b**) clinical significance of FH-associated variants and time interval for the occurrence of new CV events. Legend: LDLR—low-density cholesterol lipoprotein receptor, APOB—apolipoprotein B, PCSK9—proprotein convertase subtilisin/kexin type 9, CV—cardiovascular.

For FH patients with *LDLR, APOB,* and *PCSK9* mutations, the time interval for the occurrence of new cardiovascular events was significantly decreased, compared to FH patients without mutations (14 months vs. 27 months, *p* = 0.001) (Figure 6a). Following the stratification according to the clinical significance of FH-associated mutations, there were no significant differences in the time interval for new ASCVD occurrence (Figure 6b).

## 4. Discussion

In this study, two techniques were used to identify mutations in patients with a clinical diagnosis of FH—multiplex ligation-dependent probe amplification (MLPA) and Sanger sequencing. Since DNA sequencing cannot detect large gene rearrangements, the MLPA approach was performed [30]. The Leiden Open Source Variation Database (LOVD) is a virtual space of genetic variants, comprising 1707 unique LDLR variants since 2016, which can be accessed by all physicians in the preclinical and clinical fields [31]. However, ClinVar is a resource funded by the National Center for Biotechnology Information (NCBI), which provides a centralized database for archiving clinically relevant variants for many Mendelian pathologies, including FH [31,32]. ClinVar is a comprehensive approach to show the molecular data about patients, including many interconnected resources to improve the interpretation of the variants [31,32]. Both ClinVar and LOVD show the mutation type identified by the geneticist—pathogen/likely pathogen, benign/likely benign, or conflicting interpretations. The term “pathogen” means affecting the protein function causing disease, while “benign” indicates the lack of impairment of protein function, without causing disease [33]. However, the applicability of the term “likely” is limited to variants in which the data support a high probability of being pathogenic (>90%) or a high probability of being benign (>90%), without providing a quantitative definition of these aspects [33].

In the current study, the mutation rate registered was 37.7%, 23 patients with mutations being identified—7 patients with pathogenic/likely pathogenic mutations and 16 with polymorphisms. Regarding the pathogenic/likely pathogenic mutations, it is striking that the same mutations were targeted at exons 2, 4, and 11 of the *LDLR* gene. Therefore, further studies that include a large number of patients are recommended, targeting the identification of the genetic spectrum of FH in Romania and other populations. The difference in plasma lipid levels, even in patients bearing the same mutation, could be induced by other genetic and /or environmental factors, and the possibility of a second mutation in the LDLR gene should be considered [34].

Variants of the *LDLR*, *APOB*, *PCSK9* genes, similar to those included in the study conducted in this region of Romania, were presented by several other authors, as indicated in Table 5 [1,2,3,4,5,7,8,9,10,11,15,16,30,34,35,36,37,38,39,40,41,42,43,44].

In our study, we identified two patients with the c.81C > G mutation in exon 2 of LDLR gene with heterozygous form, which had two new CV events during the follow up—stroke and PAD. Mollaki et al. showed in a cohort study that c.1646G > A and c.1285G > A were associated with high lipid levels compared to c.858C > A and c.81C > G, which cause a milder phenotype [30]. The genotype-to-phenotype correlations revealed that receptor defective mutations cause lowered lipid levels than receptor-negative mutations of LDLR, c.81C > G being considered a defective mutation of *LDLR* [30]. The c.81C > G mutation in exon 2 of the *LDLR* gene was identified in Greece in 37 patients [30], in Switzerland in one patient [15], and in Slovakia in two patients [3] (Table 5), while in our study it was detected in two patients with heterozygous status.

The c.502G > A mutation in exon 4 of *LDLR* gene was observed in few studies—in the UK in one patient [45], in Spain in one patient [46], in the Czech Republic in one subject [47], and in Canada in one subject [1] (Table 5), while in our study we identified the same mutation in one patient with new ASCVD (stroke).

The c.1618G > A mutation in exon 11 of the *LDLR* gene was described in the study carried out by Chiou et al. in one patient from Taiwan [44], in Brazil in one patient, and in the study conducted by Jannes et al. [4], in Australia in one patient [11], in the UK in one patient [35], in Japan in one patient [34], in Greece in one patient [38], in Spain in two patients [16], and in Germany in two patients [37] (Table 5). In this study conducted in Romania, three patients with the same mutation (c.1618G > A) with homozygous status in the exon 11 of *LDLR* gene have been identified.

In our study, we recognized one patient with a pathogenic mutation—duplication of part of the *LDLR*, exhibited by the exon 13–15 duplication (c.(1845+1_1846-1)_(2311+1_2312-1)dup)). In one Italian FH patient, based on Southern blot analysis, Lelli et al. observed the insertion caused by a duplication of exons 13, 14, and 15, being the result of an unequal crossover between repetitive sequences located in intron 12 and intron 15, which was titled FH Bologna-2 [48]. Likewise, this duplication was identified in one patient in the study conducted by Futema et al., which included 48 patients with definite FH from the UK [36].

At the same time, the FH phenotype can be explained by mutations in the *APOB* and *PCSK9* genes [30], which have not been detected so far in Romania. As described above, a higher prevalence of *LDLR* variants and a lower number of variants in *APOB* and *PCSK9* were observed [49]. In our study, *APOB* and *PCSK9* mutations were synonymous, benign, and patients with these variants probably did not have a monogenic disorder of lipid metabolism, but they had a polygenic form of hypercholesterolemia. Madeira et al. reached the same conclusions in the study conducted on FH patients in Portugal [49].

Depending on the DLCN score, the subjects with possible FH had 9.8% mutations, those with probable FH had 21.3% mutations, and individuals with definite FH had 6.5% mutations, while in an FH cohort from Italy, Bertolini et al. showed that the mutation detection rate of subjects stratified according to the DLCN score was “definite FH”—91.9%, “probable FH”—76.6%, and “possible FH”—69.4% [2]. The definite FH patients had pathogenic mutations in 4.9% of cases, whereas in 8.2% of cases, no mutations were identified. The polygenic component may provide a possible explanation in this respect and future studies acknowledging these observations would be worth conducting.

Although all of the identified homozygous and heterozygous patients with pathogenic/likely pathogenic mutations were treated with lipid-lowering drugs, none of them achieved the recommended LDL–C targets (<55 mg/dL) of guidelines on dyslipidemia [24]; different therapeutic approaches are necessary in order to decrease the high cardiovascular risk of these patients [24,49].

In 38 patients included in this study, no mutations of *LDLR*, *PCSK9,* and *APOB* were detected by MLPA analysis, by exon-by-exon sequencing of amplified genomic DNA. Among patients without mutations, 8.2% had a definite FH clinical diagnosis, involving an inherited component of their disorder, which may not be monogenic or may result from more complex interactions between gene variants and the environment [35]. However, despite the addition of *LDLRAP1* and *PCSK9* to the list of genes that are associated with inherited hypercholesterolemia, there still appears to be a substantial number of patients with a clinical diagnosis of possible or defined FH who exhibit unknown genetic defect [35]. In a study that enrolled 61.217 patients, only 5.4% were diagnosed with inherited atherogenic dyslipidemia, reported primarily as a secondary diagnosis; this pathology was identified in a much smaller number of cases compared to the real number of patients, mainly because of the short period of hospitalization for acute coronary syndromes [50].

For studying genotype–phenotype interactions in a genetically homogeneous population Romanian, the analysis of the effect of mutation on the variation of plasma cholesterol levels and the expression of cardiovascular events were important, a concept also supported by Weiss et al. [37]. The pathogenic/likely pathogenic *LDLR* mutations were significant predictors of the new cardiovascular events, and the time interval for new cardiovascular events occurrence was significantly decreased, compared to FH patients without mutations. In our study, the patients with pathogenic/likely pathogenic mutations, even if they had a reduced chronic inflammation status (hsCRP with low values), expressed significantly expanded levels upon lipid profile under lipid-lowering treatment throughout the follow up, compared to patients without mutations with ASCVD. Moreover, at the end of the study, patients with pathogenic mutations recorded significantly increased cIMT and decreased EF, compared to those with ASCVD but without mutations.

Lp(a) levels are associated with a high risk of cardiovascular mortality, requiring a new pharmacotherapeutic approach, and 5–20% of patients suspected of FH had elevated Lp(a) levels [51]. In our study, we did not measure the Lp(a) values, but these findings will be exploited in a future study in which the Lp(a) of the patients will be assessed.

The statin treatment has been frequently used in our study and triple therapy has been frequently indicated for patients with pathogenic mutations. The beneficial effect of statins on cardiovascular events is due to their cholesterol-lowering properties, as inhibition of 3-hydroxy-3-methyl-glutaryl-coenzyme A (HMG–CoA) reductase can lead to pleiotropic effects. Statins have an important anti-inflammatory effect by decreasing the number of LDL particles found in the vascular wall. In addition, ezetimibe reduces the absorption of intestinal cholesterol, and along with statins, contribute to further lowering of LDLC and cardiovascular events [13].

### Study Strengths and Limitations

This is the first observational genetic approach in Romania that included patients with FH in order to identify the relationship between specific mutations in *LDLR, APOB, PCSK9*, and ASCVD. This study represents an important step in identifying cases with FH and ASCVD to create a bridge between specialties (cardiology—internal medicine—genetics). Genetic testing is essential to confirm the diagnosis of FH based on clinical and paraclinical components, despite the fact that it is not currently performed in Romania or reimbursed by the Romanian health system [52]. In the absence of any specific screening program, the percentage of undiagnosed FH carriers may be increased, leading to significant cardiovascular events. However, in practice, the diagnosis is based on the calculation of clinical risk scores related to DLCN, Simon Broome, and MedPed criteria. The clinical identification, complemented by genetic recognition of FH patients contributes to the improvement of the management and to the ASCVD decline [52]. Furthermore, these data must benefit from a special platform patient data and the results of the molecular genetics analyses should be introduced [52]. Another strength is that the same mutation on exon 11 of the *LDLR* gene (c.1618G > A) with homozygous status was identified in several patients with FH.

Nonetheless, this study had some limitations. Firstly, the methodology of the study was observational. Secondly, a small number of patients were included in the study (because for most eligible patients there were no values for EF, ABI, or lipid profile prior to their inclusion in the study). Thirdly, the current study enrolled subjects from the northeastern area of Romania, and thus, the current group of patients did not significantly reflect the entire Romanian population with ASCVD. No cascade studies were performed to examine segregation and genotype–phenotype interactions regarding the three mutations. A possible cause of the decreased frequency of LDLR mutations in our FH patients could be due to the fact that the genetic defects were benign or likely benign.

## 5. Conclusions

In this study, out of 61 FH patients included based on the DCLN score over 3, seven patients had pathogenic/likely pathogenic variants and 16 benign/likely benign variants, confirmed by MLPA and Sanger sequencing. Four pathogenic variants have been recognized, i.e., c.81C > G (exon 2 of *LDLR* gene) with heterozygous form, c.502G > A (exon 4 of *LDLR* gene) with heterozygous form, c.1618G > A (exon 11 of LDLR gene) with homozygous form, and exon 13–15 duplication (c.(1845+1_1846-1)_(2311+1_2312-1)dup)) in *LDLR* genes, which have not been reported in any study conducted in Romania. In patients with pathogenic/likely pathogenic mutations, the hsCRP and HDL–C levels were decreased, while TC and LDL–C levels were increased under lipid-lowering treatment throughout the follow up. Additionally, the pathogenic/likely pathogenic *LDLR* mutations were significant predictors of the new cardiovascular events, and the time interval for the occurrence of new cardiovascular events was significantly decreased, compared to FH patients without mutations.

## Figures and Tables

**Table 1:** Baseline characteristics of familial hypercholesterolemia (FH) patients.

Characteristics	Patients with FH				
	Overall	Without Mutation	Benign/Likely Benign Mutation	Pathogenic/Likely Pathogenic Mutation	*p*
*n*	61	38	16	7	
Age—yo (mean ± SD)	48.4 ± 12.5	50.3 ± 11.6	43.4 ± 13.6	50.1 ± 13.4	0.18
Gender (male) *n* (%)	22 (36.1%)	14 (36.8%)	5 (31.3%)	3 (42.9%)	0.86
Smoker *n* (%)	18 (29.5 %)	12 (31.6%)	5 (31.3%)	1(14.3%)	0.64
High blood pressure *n* (%)	31 (50.8 %)	22 (57.9%)	6 (37.5%)	3(42.9%)	0.35
CHD history *n* (%)	13 (21.3%)	10 (26.3%)	3 (18.8%)	0	0.06
PAD history *n* (%)	9 (14.8%)	7 (18.4%)	2 (12.5%)	0	
CHD + PAD history *n* (%)	14 (23%)	6 (15.8%)	3 (18.8%)	5 (71.4%)	
Obesity *n* (%)	22 (36.1%)	14(36.8%)	4 (25%)	4 (57.4%)	0.33
Type 2 diabetes *n* (%)	8 (13.1%)	5 (13.2%)	2 (12.5%)	1 (14.3%)	0.99
Physical inactivity *n* (%)	30 (49.2%)	21 (55.3%)	5 (31.3%)	4 (57.1%)	0.25
TC mg/dL (median ± IQR)	315 ± 56	307.5 ± 44	320 ± 41	353 ± 206	0.02 *
LDL–C mg/dL (mean ± SD)	254.2 ± 53	246.2 ± 46.2	255.4 ± 46.5	294.7 ± 85.1	0.31
HDL–C mg/dL (median ± IQR)	45.8 ± 18	45 ± 12.3	48.5 ± 16.1	39 ± 16.2	0.28
TG mg/dL (mean ± SD)	174.4 ± 92	179.5 ± 92.2	160.6 ± 102.3	178.9 ± 74.2	0.61
hsCRP mg/L (mean ± SD)	5.85 ± 2.29	5.8 ± 2.2	6.3 ± 2.2	7.4 ± 2.4	0.26
ECG changes *n* (%)	25 (41%)	13 (34.2%)	7 (43.8%)	5 (71.4%)	0.18
EF % (mean ± SD)	53.2 ± 9.8	53.8 ± 9.4	54.1 ± 9.6	47.6 ± 11.8	0.35
ABI (mean ± SD)	0.96 ± 0.93	0.85 ± 0.07	0.85 ± 0.08	0.77 ± 0.11	0.15
cIMT mm (mean ± SD)	0.95 ± 0.33	0.91 ± 0.32	0.93 ± 0.36	1.21 ± 0.31	0.09
Lipid-Lowering Agents					
Statin *n* (%)	22 (36.1%)	14 (36.8%)	6 (37.5%)	2 (28.6%)	0.41
Statin + ezetimibe *n* (%)	18 (29.5%)	9 (23.7%)	7 (43.8%)	2 (28.6%)	
Statin + fenofibrate *n* (%)	8 (13.1 %)	7 (18.4%)	1 (6.3%)	0	
Statin + ezetimibe + fenofibrate *n* (%)	13 (21.3%)	8 (21.1%)	2 (12.5%)	3 (42.9%)	
DLCN Score (mean ± SD)	6.4 ± 2.9	5.9 ± 2.5	6.2 ± 1.9	9.6 ± 4.9	0.02 *

Legend: CHD—coronary heart disease, PAD—peripheral arterial disease, TC—total cholesterol, LDL–C—low-density cholesterol lipoprotein, HDL–C—high-density cholesterol lipoprotein, TG—triglycerides, hsCRP—high-sensitivity C-reactive protein, ECG—electrocardiogram, EF—ejection fraction, ABI—ankle–brachial index, cIMT—carotid intima–media thickness, DLCN—Dutch Lipid Clinic Network, * *p* < 0.05.

**Table 2:** *LDLR, PCSK9*, and *APOB* variants identified in the Romania patients.

Gene	Location	Nucleotide Change	Protein Change	Number of Carriers
Pathogenic Variants				
*LDLR*	Exon 2	c.81C > G	p.(Cys27Trp)	2
*LDLR*	Exon 4	c.502G > A	p.(Asp168Asn)	1
*LDLR*	Exon 11	c.1618G > A	p.(Ala540Thr)	3
*LDLR*	Exon 13–15	c.(1845+1_1846-1)_(2311+1_2312-1)dup	p (?)	1
Benign Variants				
*LDLR*	Exon 2	c.81C > T	p.(Cys27=)	3
*LDLR*	Exon 10	c.1413A > G	p.(Arg471=)	15
*LDLR*	Exon 11	c.1617C > T	p.(Pro539=)	3
*LDLR*	Exon 12	c.1773C > T	p.(Asn591=)	12
*LDLR*	Exon 13	c.1959T > C	p.(Val653=)	12
*LDLR*	Exon 15	c.2232A > G	p.(Arg744=)	14
*APOB*	Exon 26	c.10740C > T	p.(Asn3580=)	3
Conflicting interpretations				
*LDLR*	Exon 3	c.211G > A	p.(Gly71Arg)	1
*LDLR*	Exon 7	c.1060+7=	p (?)	18
*LDLR*	Intron 7	c.1060+10G > A	p (?)	8
*PCSK9*	Exon 7	c.1026A > G	p.(Gln342=)	6

Legend: *LDLR*—low-density lipoprotein receptor, *APOB*—apolipoprotein B; *PCSK9*—proprotein convertase subtilisin/kexin type 9 Variants were classified as pathogenic/likely pathogenic, benign/likely benign, and of conflicting interpretations according to ClinVar and Leiden Open Source Variation Database (LVOD).

**Table 3:** The characteristics of FH patients according to cardiovascular events.

Characteristics	Patients with FH					
	ASCVD- Mutation-	ASCVD+ Mutation-	ASCVD- Benign/Likely Benign Mutation+	ASCVD+ Benign/Likely Benign Mutation+	ASCVD+ Pathogenic/Likely PATHOGENIC Mutation+	*p*
*n* (%)	25 (41%)	13 (21.31%)	8 (13.1%)	8 (13.1%)	7 (11.5%)	
TC baseline mg/dL (median ± IQR)	312.7 ± 20.4	352.8 ± 63.2	323.9 ± 26.9	352.6 ± 42.2	445.6 ± 203.9	0.01 *
TC 12 mo mg/dL (median ± IQR)	249.1 ± 17.9	284.5 ± 51.8	261 ± 13.9	278.5 ± 44.2	310.3 ± 63.5	0.009 *
TC 24 mo mg/dL (median ± IQR)	227.7 ± 19.3	254.8 ± 47.4	236.1 ± 12.9	256.6 ± 29.8	278.7 ± 57.9	0.01 *
TC 36 mo mg/dL (median ± IQR)	210.8 ± 17.1	230.9 ± 14.3	229.3 ± 12.2	245.6 ± 29.6	271.9 ± 53.3	0.001 *
LDL–C baseline mg/dL (mean ± SD)	233.4 ± 29.1	270.8 ± 62.5	240.1 ± 29.2	257.8 ± 54.7	309.7 ± 78.7	0.15
LDL–C 12 mo mg/dL (mean ± SD)	163.6 ± 21.4	204.4 ± 50.9	166.6 ± 13.3	195.4 ± 52.9	234.1 ± 66.7	0.002 *
LDL–C 24 mo mg/dL (mean ± SD)	137.4 ± 17.8	167.5 ± 47.6	139.6 ± 14.2	168.5 ± 39.7	202.4 ± 59.7	0.003 *
LDL–C 36 mo mg/dL (mean ± SD)	113.1 ± 17.3	146.5 ± 11.6	131.1 ± 14.9	159.1 ± 35.1	189.3 ± 56.5	0.001 *
HDL–C baseline mg/dL (median ± IQR)	51.3 ± 12.5	42.4 ± 9.8	54.5 ± 16.4	55.5 ± 19.9	43.1 ± 7.9	0.14
HDL–C 12 mo mg/dL (median ± IQR)	61.7 ± 8.9	53.9 ± 9.8	64.3 ± 12.1	64.4 ± 12.4	54.9 ± 10.1	0.19
HDL–C 24 mo mg/dL (median ± IQR)	67.4 ± 7.9	62.4 ± 7.1	71.1 ± 6.8	68.4 ± 9.4	59.4 ± 10.8	0.05 *
HDL–C 36 mo mg/dL (median ± IQR)	75.1 ± 8.2	61.6 ± 7.7	73.1 ± 7.9	65.1 ± 4.8	58.3 ± 5.8	0.001 *
TG baseline mg/dL (mean ± SD)	166.8 ± 90.9	203.7 ± 93.3	204.1 ± 128.1	140.1 ± 80.2	152.4 ± 47.9	0.19
TG 12 mo mg/dL (mean ± SD)	124.2 ± 48.8	142.4 ± 51.3	151.6 ± 69.4	109.1 ± 39.7	124.7 ± 29.7	0.26
TG 24 mo mg/dL (mean ± SD)	116.5 ± 31.2	124.9 ± 30.4	125.1 ± 36.3	106.8 ± 34.3	122.4 ± 30.5	0.41
TG 36 mo mg/dL (mean ± SD)	120.6 ± 22.3	123.1 ± 22.3	126.1 ± 28.5	107.1 ± 33.1	121.7 ± 25.4	0.25
hsCRP baseline mg/L (mean ± SD)	5.1 ± 1.9	7.3 ± 1.8	6.1 ± 2.7	6.7 ± 1.6	7.1 ± 2.5	0.02 *
hsCRP 12 mo mg/L (mean ± SD)	4.1 ± 1.8	6.1 ± 1.3	4.8 ± 1.9	5.7 ± 1.6	5.8 ± 2.4	0.02 *
hsCRP 24 mo mg/L (mean ± SD)	3.5 ± 1.4	5.3 ± 1.3	4.1 ± 1.6	4.4 ± 1.7	4.7 ± 2.3	0.01 *
hsCRP 36 mo mg/L (mean ± SD)	0.6 ± 0.2	7.2 ± 1.2	2.4 ± 2.7	5.9 ± 1.1	6.7 ± 1.8	0.001 *
EF baseline % (mean ± SD)	56.4 ± 7.6	48.7 ± 10.5	53.8 ± 8.6	49.4 ± 10.8	53.3 ± 12.9	0.13
EF 12 mo % (mean ± SD)	55.4 ± 6.6	48.1 ± 9.6	52.5 ± 7.6	48.8 ± 8.4	50.7 ± 14.8	0.11
EF 24 mo % (mean ± SD)	54.8 ± 6.3	43.1 ± 10.7	52.5 ± 7.6	47.5 ± 8.1	44.3 ± 16. 2	0.006 *
EF 36 mo % (mean ± SD)	53.8 ± 6.7	41.2 ± 12.1	51.9 ± 7.5	45 ± 10.4	37.9 ± 17.3	0.002 *
ABI baseline (mean ± SD)	0.86 ± 0.06	0.81 ± 0.06	0.89 ± 0.08	0.79 ± 0.06	0.78 ± 0.11	0.01 *
ABI 12 mo (mean ± SD)	0.89 ± 0.05	0.85 ± 0.05	0.91 ± 0.06	0.81 ± 0.06	0.81 ± 0.08	0.001 *
ABI 24 mo (mean ± SD)	0.94 ± 0.07	0.89 ± 0.71	0.94 ± 0.08	0.84 ± 0.13	0.85 ± 0.07	0.004 *
ABI 36 mo (mean ± SD)	0.94 ± 0.04	0.82 ± 0.11	0.94 ± 0.03	0.81 ± 0.09	0.83 ± 0.09	0.001 *
cIMT baseline mm (mean ± SD)	0.82 ± 0.28	1.08 ± 0.32	0.89 ± 0.36	1.13 ± 0.31	1.04 ± 0.42	0.03 *
cIMT 12 mo mm (mean ± SD)	0.75 ± 0.23	1.03 ± 0.25	0.81 ± 0.31	1.05 ± 0.24	1.06 ± 0.31	0.002 *
cIMT 24 mo mm (mean ± SD)	0.74 ± 0.25	0.99 ± 0.31	0.75 ± 0.29	0.96 ± 0.31	0.94 ± 0.36	0.01 *
cIMT 36 mo mm (mean ± SD)	0.73 ± 0.15	1.07 ± 0.18	0.76 ± 0.12	1.08 ± 0.21	1.13 ± 0.15	0.001 *

Legend: ASCVD- atherosclerotic cardiovascular disease, TC—total cholesterol, LDL–C—low-density cholesterol lipoprotein, HDL–C—high-density cholesterol lipoprotein, TG—triglycerides, hsCRP—high-sensitivity C-reactive protein, EF—ejection fraction, ABI—ankle–brachial index, cIMT—carotid intima–media thickness, mo-months * *p* < 0.05.

**Table 4:** Independent factors for cardiovascular events in FH patients.

Variable	B	SE	Wald	df	*p*	OR	95.0% CI for HR	
							Lower	Upper
Type of mutations Pathogenic Mutations Benign Mutations			5.41	2	0.05 *			
	1.57	0.68	5.29	1	0.02 *	4.81	1.26	18.32
	0.37	0.48	0.61	1	0.44	1.45	0.57	3.73
EF baseline	0.01	0.03	0.05	1	0.82	1.01	0.95	1.08
cIMT baseline	0.33	1.12	0.08	1	0.77	1.39	0.15	13.29
ABI baseline	−3.96	4.74	0.69	1	0.41	0.02	0.001	20.73
hsCRP baseline	0.16	0.13	1.51	1	0.22	1.18	0.91	1.53
LDL–C baseline	0.04	0.08	0.33	1	0.57	1.01	0.99	1.02
DLCN Score Possible FH Probable FH Definite FH
	ref	ref	1.69 ref	2 ref	0.43 ref	ref	ref	ref
	0.48	0.66	0.53	1	0.47	1.62	0.45	5.87
	−0.39	1.51	0.07	1	0.79	0.67	0.04	12.94
Simon Broome score	0.56	0.73	0.59	1	0.44	1.75	0.42	7.37
Lipid-lowering DrugsStatin Statin + Ezetimibe Statin + Fenofibrate Statin + Fenofibrate + Ezetimibe
	ref	ref	1.35 ref	3 ref	0.72 ref	ref	ref	ref
	0.15	0.61	0.06	1	0.81	1.17	0.35	3.84
	0.46	0.76	0.37	1	0.54	1.59	0.36	7.09
	−0.44	0.67	0.44	1	0.51	0.64	0.18	2.36

Legend: LDL–C—low-density cholesterol lipoprotein, hsCRP—high-sensitivity C-reactive protein, EF—ejection fraction, ABI—ankle–brachial index, DLCN-Dutch Lipid Clinic Network, cIMT—carotid intima–media thickness, OR odd ratio * *p* < 0.05.

**Table 5:** Characteristics of different studies for genotypes of the FH patients.

Locality	Country	Diagnostic Criteria	Number of Patients	Number of Patients with Mutations	Technique- Molecular Analysis	Gene	Number of Detected Mutations
Western Europe	Italy [2]	DLCN	1018	94	MLPAN orthern blot analysis and RT-PCR amplification In silico analysis	*LDLR* *APOB* *PCSK9*	984 *LDLR* 22 *APOB* 2 *PCSK9*
	Switzerland [15]	LDL–C 95th percentile	94	NA	NGS (Illumina) Sanger sequencing	*LDLR* *APOB* *PCSK9*	43 *LDLR* 5 *APOB*3 6 *PCSK9*
	UK-1 [45]	SB criteria	791	134	SSCP analysis	*LDLR*	51 *LDLR*
	UK-2 [35]	SB criteria	280	171 He FH patients 28 Ho/ compound He	MLPA sequencing of amplified fragments of genomic DNA or mRNA	*LDLR* *LDLRAP1* *PCSK9* *APOB*	98 *LDLR* 2 *PCSK9* 5 *LDLRAP1* 14 *APOB*
	UK-3 [36]	SB FH register SB criteria	48	14	MLPA NGS (Illumina) Sanger sequencing	*LDLR* *LDLRAP1* *PCSK9* *APOB*	17 *LDLR* 1 *LDLRAP1* 2 *PCSK9* 3 *APOB*
	Spain [16]	Spanish FH Registry	476	329	SSCP analysis	*LDLR* *APOB*	116 *LDLR* 4 *APOB*
	Germany [37]	LDL–C 90th percentile	162	27	MLPA Direct sequencing on LDLR gene	*LDLR*	24 *LDLR*
	Portugal (Azores Island) [5]	SB	33	33	LIPOchip^®^ Array version 7 (DNA array) Direct sequencing for exons 2–6	*LDLR*	18 *LDLR*
Central and Eastern Europe	Slovakia [3]	LDL–C 95th percentile + HCH in family	359	16 for APOB 164 for LDLR	TaqMan SNP Genotyping Assay ID Bidirectional sequencing on LDLR gene MLPA	*LDLR* *APOB*	54 *LDLR* 1 *APOB*
	Greece-1 [38]	LDL–C 95th percentile CVD history CVD family history tendon xanthomas	183	78	DGGE analysis	*LDLR* *APOB*	17 *LDLR* 0 *APOB*
	Greece-2 [30]	HeFH	561	140	DNA sequencing of the LDLR gene	*LDLR*	26 *LDLR*
	Russia 1 [39]	DLCN	80	80	Sanger sequencing In silico analysis	*LDLR* *APOB*	12 *LDLR* 0 *APOB*
	Russia 2 [40]	LDL–C 95th percentile CVD history CVD family history tendon xanthomas	45	24	Automated DNA sequencing	*LDLR* *APOB*	21 *LDLR* 0 *APOB*
	Poland-1 [41]	LDL–C 90th percentile	30 families	17 families	SSCP analysis sequencing of polymerase chain reaction restriction enzyme patterns on Southern blots and long-PCR	*LDLR* *APOB*	11 *LDLR* 1 *APOB*
	Poland-2 [9]	SB	161	40	High resolution melt Direct sequencing MLPA	*LDLR* *APOB*	39 *LDLR* 1 *APOB*
	Czech Republic [47]	personal history and/or family history of premature CHD elevated TC, LDL95th percentile	3914	1296	denaturing high-performance liquid chromatography (dHPLC) PCR-RFLP Sanger sequencing MLPA	*LDLR* *APOB*	864 *LDLR* 32 *APOB*
Worldwide	Canada [1]	DLCN The British Columbia FH Registry	626	275	NGS (Illumina)	*LDLR* *APOB* *PCSK9* *LDLRAP1*	131 unique FH-causing SNVs 38 CNV *LDLR* 0 CNV *PCSK9*, *APOB*
	Brazil [4]	DLCN SB	248	125	MLPA	*LDLR* *APOB* *PCSK9*	71 *LDLR* 2 *APOB* 0 *PCSK9*
	Colombia [42]	MedPed	24 families	NA	Sanger sequencing	*LDLR*	18 *LDLR* 3 pathogenic *LDLR*
	Australia [11]	mutations previously determined	30	NA	Ion Torrent Personal Genome Machine (PGM) sequencing Sanger sequencing MLPA	*LDLR*	2179 *LDLR*
	Malaysia [10]	SB	164	117	Denaturing High-Performance Liquid Chromatography MLPA In silico analyses of variant effects	*LDLR*	8 mutation *LDLR*21 variants
	Sri Lanka [7]	Modified SB DCLN	27	5	Sanger sequencing	*LDLR*	4 variants He 1 mutation He compound
	Saudi Arabia [43]	DLCN	2	2	Sanger sequencing	*LDLR* *APOB* *PCSK9*	2 *LDLR* mutations 0 *APOB* 0 *PCSK9*
	Iran [8]	SB	80	NA	ARMS-PCR PCR- RFLP assay	*LDLR* *APOB* *PCSK9*	2 *LDLR* mutations 6 *LDLR* polymorphism 0 *APOB* 0 *PCSK9*
	Taiwan [44]	SB	125	76	Microarray resequencing Sanger sequencing	*LDLR* *APOB*	66 *LDLR* mutations 10 *APOB* mutations
	Japan [34]	criteria suggested by the Japan Atherosclerosis Society	205	118	SSCP assay MLPA	*LDLR*	53 *LDLR* mutations 21 large rearrangements

Legend: *LDLR*—low-density lipoprotein receptor, *APOB*—apolipoprotein B; *PCSK9*—proprotein convertase subtilisin/kexin type 9, DLCN—Dutch Lipid Clinic Network, HeFH—heterozygous form of familial hypercholesterolemia, SB- Simon Broome, MLPA-multiplex ligation-dependent probe amplification, RT PCR—reverse transcription-polymerase chain reaction, NGS—next-generation sequencing, SSCP—single-strand conformation polymorphism, ARMS–PCR—tetra-primer amplification refractory mutation system–polymerase chain reaction, PCR–RFLP—restriction fragment length polymorphism, DDGE—denaturing gradient gel electrophoresis.

## Data Availability

Not applicable.

## Supplementary Materials

The following are available online at https://www.mdpi.com/article/10.3390/jcm10071399/s1, Table S1: *LDLR*, *APOB*, and *PCSK9* variants identified in the Romanian FH cohort and not reported previously in this population.
