# Supplementary material for: Molecular Genetic Approach and Evaluation of Cardiovascular Events in Patients with Clinical Familial Hypercholesterolemia Phenotype from Romania
Source: J Clin Med. 2021 Mar 31;10(7):1399. doi: 10.3390/jcm10071399 (PMC8036385; doi:10.3390/jcm10071399)
Supplement: Supplementary file 1 [file jcm-10-01399-s001.pdf]

Table S1. LDLR, APOB and PCSK9 variants identified in the Romanian FH cohort and not reported previously in this population.
REFERENCE SEQUENCE: NM_000527.5 for LDLR, NM_000384.3 for APOB, NM_174936.3 for PCSK9

ID	GENE	EXON /INTRON	DOMAIN	cDNA change	Protein change	dbSNP ID	ClinVar assertion	LOVD/HGMD annotation and assertion	Sanger confirmation	CV events
H7	LDLR<br>LDLR	10<br>11	EGF precursor<br>LDLR class B4	c.1413A>G<br>c.1617C>T	p.(Arg471=)<br>p.(Pro539=)	rs5930 He<br>rs5929 He	Benign<br>Benign	Benign<br>Benign	YES	YES<br>Stroke
H8	LDLR<br>LDLR<br>LDLR	11<br>12<br>13	LDLR class B4<br>LDLR class B5<br>LDLR class B6	c.1617C>T<br>c.1773C>T<br>c.1959T>C	p.(Pro539=)<br>p.(Asn591=)<br>p.(Val653=)	rs5929<br>rs688 Ho<br>rs5925 Ho	Benign<br>Likely benign<br>Likely benign	Benign<br>Benign<br>Benign	YES	YES<br>Stroke
H10	LDLR<br>LDLR	7<br>7i	NA<br>Intron	c.1060+7=<br>c.1060+10G>A	NA<br>NA	rs2738442<br>rs12710260 Ho	Conflicting interpretations<br>Conflicting interpretations	No referenced<br>Conflicting interpretations	YES	NO
H14	LDLR<br>LDLR	12<br>13	LDLR class B5<br>LDLR class B6	c.1773C>T<br>c.1959T>C	p.(Asn591=)<br>p.(Val653=)	rs5929<br>rs5925 He	Benign<br>Likely benign	Benign<br>Benign	YES	NO
H15	LDLR<br>LDLR	7<br>7i	NA<br>Intron	c.1060+7=<br>c.1060+10G>A	NA<br>NA	rs2738442<br>rs12710260 Ho	Conflicting interpretations<br>Conflicting interpretations	No referenced<br>Conflicting interpretations	YES	NO
H16	LDLR<br>LDLR	12<br>13	LDLR class B5<br>LDLR class B6	c.1773C>T<br>c.1959T>C	p.(Asn591=)<br>p.(Val653=)	rs5929<br>rs5925 He	Benign<br>Likely benign	Benign<br>Benign	YES	NO
H18	LDLR<br>LDLR<br>LDLR<br>LDLR<br>LDLR<br>LDLR<br>LDLR	2<br>7<br>7i<br>10<br>12<br>13<br>15	ligand-binding<br>NA<br>Intron<br>LDLR class B2<br>LDLR class B5<br>LDLR class B6<br>clustered O-linked oligosaccharides	c.81C>G<br>c.1060+7=<br>c.1060+10G>A<br>c.1413A>G<br>c.1773C>T<br>c.1959T>C<br>c.2232A>G	p.(Cys27Trp)<br>NA<br>NA<br>p.(Arg471=)<br>p.(Asn591=)<br>p.(Val653=)<br>p.(Arg744=)	NA<br>rs2738442<br>rs12710260 Ho<br>rs5930<br>rs688 Ho<br>rs5925 Ho<br>rs5927 Ho	Likely pathogenic<br>Conflicting interpretations<br>Benign<br>Likely benign<br>Benign<br>Likely benign<br>Benign	Probably pathogenic<br>No referenced<br>Conflicting interpretations<br>Benign<br>Benign<br>Benign<br>Benign	YES	YES<br>Stroke
H19	LDLR<br>LDLR<br>LDLR<br>LDLR<br>PCSK9	7<br>10<br>11<br>12<br>7	NA<br>LDLR class B2<br>LDLR class B4<br>LDLR class B5<br>NA	c.1060+7=<br>c.1413A>G<br>c.1618G>A<br>c.1773C>T<br>c.1026A>G	NA<br>p.(Arg471=)<br>p.(Ala540Thr)<br>p.(Asn591=)<br>p.(Gln342=)	rs2738442<br>rs5930<br>NA<br>rs688 Ho<br>rs509504 Ho	Conflicting interpretations<br>Benign<br>Pathogenic<br>Likely benign<br>Conflicting interpretations	No referenced<br>Benign<br>Likely pathogenic<br>Benign<br>No referenced	YES	YES<br>ACS
H24	LDLR<br>LDLR	7<br>15	NA<br>clustered O-linked oligosaccharides	c.1060+7=<br>c.2232A>G	NA<br>p.(Arg744=)	rs2738442<br>rs5927 Ho	Conflicting interpretations<br>Benign	No referenced<br>Benign	YES	YES<br>PAD
H25	LDLR	15	clustered O-linked	c.2232A>G	p.(Arg744=)	rs5927 Ho	Benign	Benign	YES	NO

			oligosaccharides							
H26	PCSK9<br>APOB	7<br>26	NA<br>NA	c.1026A>G<br>c.10740C>T	p.(Gln342=)<br>p.(Asn3580=)	rs509504 Ho<br>rs150312765	Conflicting interpretations<br>Benign	No referenced<br>No referenced	YES	YES<br>PAD
H32	LDLR<br>LDLR<br>LDLR<br>LDLR<br>LDLR<br><br>PCSK9	7<br>10<br>12<br>13<br>15<br><br>7	NA<br>LDLR class B2<br>LDLR class B5<br>LDLR class B6<br>clustered O-linked<br>oligosaccharides<br>NA	c.1060+7=<br>c.1413A>G<br>c.1773C>T<br>c.1959T>C<br>c.2232A>G<br><br>c.1026A>G	NA<br>p.(Arg471=)<br>p.(Asn591=)<br>p.(Val653=)<br>p.(Arg744=)<br><br>p.(Gln342=)	rs2738442<br>rs5930<br>rs688 He<br>rs5925 He<br>rs5927 He<br><br>rs509504 Ho	Conflicting interpretations<br>Benign<br>Likely benign<br>Likely benign<br>Benign<br><br>Conflicting interpretations	No referenced<br>Benign<br>Benign<br>Benign<br>Benign<br><br>No referenced	YES	YES<br>Stroke
H40	LDLR<br>LDLR<br>LDLR<br>LDLR<br>LDLR<br>PCSK9	7<br>7i<br>10<br>12<br>13<br>7	NA<br>Intron<br>LDLR class B2<br>LDLR class B5<br>LDLR class B6<br>NA	c.1060+7=<br>c.1060+10G>A<br>c.1413A>G<br>c.1773C>T<br>c.1959T>C<br>c.1026A>G	NA<br>NA<br>p.(Arg471=)<br>p.(Asn591=)<br>p.(Val653=)<br>p.(Gln342=)	rs2738442<br>rs12710260 He<br>rs5930<br>rs688 He<br>rs5925 He<br>rs509504 Ho	Conflicting interpretations<br>Conflicting interpretations<br>Benign<br>Likely benign<br>Likely benign<br>Conflicting interpretations	No referenced<br>Conflicting interpretations<br>Benign<br>Benign<br>Benign<br>No referenced	YES	YES<br>ACS
H41	LDLR<br>LDLR<br>LDLR<br>LDLR<br>LDLR<br>LDLR<br><br>LDLR	7<br>7i<br>10<br>12<br>13<br>15<br><br>12i_15i	NA<br>NA<br>LDLR class B2<br>LDLR class B5<br>LDLR class B6<br>clustered O-linked<br>oligosaccharides<br>A750GfsX29; FH Bologna-2	c.1060+7=<br>c.1060+10G>A<br>c.1413A>G<br>c.1773C>T<br>c.1959T>C<br>c.2232A>G<br><br>c.(1845+1_1846-1)_(2311+1_2312-1)dup	NA<br>NA<br>p.(Arg471=)<br>p.(Asn591=)<br>p.(Val653=)<br>p.(Arg744=)<br><br>exon 13-15	rs2738442<br>rs12710260<br>rs5930<br>rs688 He<br>rs5925 He<br>rs5927 He	Conflicting interpretations<br>Conflicting interpretations<br>Benign<br>Likely benign<br>Likely benign<br>Benign<br><br>Pathogenic	No referenced<br>Conflicting interpretations<br>Benign<br>Benign<br>Benign<br>Benign<br><br>Pathogenic	YES	YES<br>Stroke
H44	LDLR<br>LDLR<br>LDLR<br>LDLR<br>LDLR	2<br>7<br>10<br>11<br>15	LDLR class A1<br>NA<br>LDLR class B2<br>LDLR class B4<br>clustered O-linked<br>oligosaccharides	c.81C>T<br>c.1060+7=<br>c.1413A>G<br>c.1617C>T<br>c.2232A>G	p.(Cys27=)<br>NA<br>p.(Arg471=)<br>p.(Pro539=)<br>p.(Arg744=)	rs2228671<br>rs2738442<br>rs5930<br>rs5929 Ho<br>rs5927 Ho	Benign<br>Conflicting interpretations<br>Benign<br>Benign<br>Benign	Benign<br>No referenced<br>Benign<br>Benign<br>Benign	YES	YES<br>ACS
H46	LDLR<br>LDLR<br>LDLR<br>LDLR	7<br>7i<br>10<br>11	NA<br>Intron<br>LDLR class B2<br>LDLR class B4	c.1060+7=<br>c.1060+10G>A<br>c.1413A>G<br>c.1618G>A	NA<br>NA<br>p.(Arg471=)<br>(p.Ala540Thr)	rs2738442<br>rs12710260 He<br>rs5930<br>NA	Conflicting interpretations<br>Conflicting interpretations<br>Benign<br>Pathogenic	No referenced<br>Conflicting interpretations<br>Benign<br>Likely pathogenic	YES	YES<br>ACS

	LDLR	12	LDLR class B5	c.1773C>T	p.(Asn591=)	rs688 He	Likely benign	Benign		
	LDLR	13	LDLR class B6	c.1959T>C	p.(Val653=)	rs5925 He	Likely benign	Benign		
	LDLR	15	clustered O-linked oligosaccharides	c.2232A>G	p.(Arg744=)	rs5927 He	Benign	Benign		
H51	LDLR	3	NA	c.211G>A	p.(Gly71Arg)	rs766903209	Conflicting interpretations	No referenced	YES	YES<br>ACS
	LDLR	7	NA	c.1060+7=	NA	rs2738442	Conflicting interpretations	No referenced		
	LDLR	10	LDLR class B2	c.1413A>G	p.(Arg471=)	rs5930	Benign	Benign		
	LDLR	13	LDLR class B6	c.1959T>C	p.(Val653=)	rs5925 He	Likely benign	Benign		
	LDLR	15	clustered O-linked oligosaccharides	c.2232A>G	p.(Arg744=)	rs5927 He	Benign	Benign		
	APOB	26	NA	c.10740C>T	p.(Asn3580=)	rs150312765	Benign	No referenced		
H52	LDLR	2	ligand-binding	c.81 C>G	p.(Cys27Trp)	NA	Likely pathogenic	Likely pathogenic	YES	YES<br>PAD
	LDLR	7	NA	c.1060+7=	NA	rs2738442	Conflicting interpretations	No referenced		
	LDLR	10	LDLR class B2	c.1413A>G	p.(Arg471=)	rs5930	Benign	Benign		
	LDLR	12	LDLR class B5	c.1773C>T	p.(Asn591=)	rs688 He	Likely benign	Benign		
	LDLR	13	LDLR class B6	c.1959T>C	p.(Val653=)	rs5925 He	Likely benign	Benign		
	LDLR	15	clustered O-linked oligosaccharides	c.2232A>G	p.(Arg744=)	rs5927 He	Benign	Benign		
	PCSK9	7	NA	c.1026A>G	p.(Gln342=)	rs509504 Ho	Conflicting interpretations	No referenced		
	APOB	26	NA	c.10740C>T	p.(Asn3580=)	rs150312765	Benign	No referenced		
H53	LDLR	7	NA	c.1060+7=	NA	rs2738442	Conflicting interpretations	No referenced	YES	YES<br>ACS
	LDLR	10	LDLR class B2	c.1413A>G	p.(Arg471=)	rs5930	Benign	Benign		
	LDLR	11	LDLR class B4	c.1618G>A	p.(Ala540Thr)	NA	Pathogenic	Pathogenic		
	LDLR	15	clustered O-linked oligosaccharides	c.2232A>G	p.(Arg744=)	rs5927 He	Benign	Benign		
	PCSK9	7	NA	c.1026A>G	p.(Gln342=)	rs509504 Ho	Conflicting interpretations	No referenced		
H54	LDLR	4	LDLR class A4	c.502G>A	p.(Asp168Asn)	rs200727689	Pathogenic	Likely pathogenic	YES	YES<br>Stroke
	LDLR	7	NA	c.1060+7=	NA	rs2738442	Conflicting interpretations	No referenced		
	LDLR	10	LDLR class B2	c.1413A>G	p.(Arg471=)	rs5930	Benign	Benign		
	LDLR	15	clustered O-linked oligosaccharides	c.2232A>G	p.(Arg744=)	rs5927 He	Benign	Benign		
H55	LDLR	2	LDLR class A1	c.81C>T	p.(Cys27=)	rs2228671	Benign	Benign	YES	NO
	LDLR	7	NA	c.1060+7=	NA	rs2738442	Conflicting interpretations	No referenced		
	LDLR	7i	Intron	c.1060+10G>A	NA	rs12710260 He	Conflicting interpretations	Conflicting interpretations		
	LDLR	10	LDLR class B2	c.1413A>G	p.(Arg471=)	rs5930	Benign	Benign		
	LDLR	15	clustered O-linked	c.2232A>G	p.(Arg744=)	rs5927 Ho	Benign	Benign		

			oligosaccharides							
H56	LDLR<br>LDLR<br>LDLR<br>LDLR<br>LDLR	7<br>10<br>12<br>13<br>15	NA<br>LDLR class B2<br>LDLR class B5<br>LDLR class B6<br>clustered O-linked oligosaccharides	c.1060+7=<br>c.1413A>G<br>c.1773C>T<br>c.1959T>C<br>c.2232A>G	NA<br>p.(Arg471=)<br>p.(Asn591=)<br>p.(Val653=)<br>p.(Arg744=)	rs2738442<br>rs5930<br>rs688 He<br>rs5925 He<br>rs5927 Ho	Conflicting interpretations<br>Benign<br>Likely benign<br>Likely benign<br>Benign	No referenced<br>Benign<br>Benign<br>Benign<br>Benign	YES	NO
H57	LDLR<br>LDLR<br>LDLR<br>LDLR<br>LDLR<br>LDLR<br>LDLR	2<br>7<br>7i<br>10<br>12<br>13<br>15	LDLR class A1<br>NA<br>Intron<br>LDLR class B2<br>LDLR class B5<br>LDLR class B6<br>clustered O-linked oligosaccharides	c.81C>T<br>c.1060+7=<br>c.1060+10G>A<br>c.1413A>G<br>c.1773C>T<br>c.1959T>C<br>c.2232A>G	p.(Cys27=)<br>NA<br>NA<br>p.(Arg471=)<br>p.(Asn591=)<br>p.(Val653=)<br>p.(Arg744=)	rs2228671<br>rs2738442<br>rs12710260<br>rs5930<br>rs688 He<br>rs5925 He<br>rs5927 Ho	Benign<br>Conflicting interpretations<br>Conflicting interpretations<br>Benign<br>Likely benign<br>Likely benign<br>Benign	Benign<br>No referenced<br>Conflicting interpretations<br>Benign<br>Benign<br>Benign<br>Benign	YES	YES<br>PAD

*Words written in red signify pathogenic mutations
